# The New Era of Long-Range “Zero-Interception” Ambient Backscattering Systems: 130 m with 130 nA Front-End Consumption

**DOI:** 10.3390/s22114151

**Published:** 2022-05-30

**Authors:** Spyridon Nektarios Daskalakis, Apostolos Georgiadis, Manos M. Tentzeris, George Goussetis, George Deligeorgis

**Affiliations:** 1 School of Engineering & Physical Sciences, Institute of Sensors, Signals and Systems, Heriot-Watt University, Edinburgh EH14 4AS, UK; g.goussetis@hw.ac.uk; 2 Independent Researcher, 2582 CN The Hague, The Netherlands; apostolos.georgiadis@ieee.org; 3 School of Electrical and Computer Engineering, Georgia Institute of Technology, Atlanta, GA 30332, USA; etentze@ece.gatech.edu; 4 Institute of Electronic Structure and Laser (IESL), Foundation for Research and Technology—Hellas (FORTH), 70013 Heraklion, Greece; deligeo@physics.uoc.gr

**Keywords:** backscatter communication, Internet of Things (IoT), low power communications, radio frequency (RF) identification (RFID) sensors, software defined radios (SDRs)

## Abstract

Internet of Things applications based on backscatter radio principles have appeared to address the limitations of high cost and high power consumption. While radio-frequency identification (RFID) sensor nodes are among the most commonly utilized state-of-the-art technologies, their range for passive implementations is typically short and well below 10 m being impractical for “rugged” applications where approaching the tag at such proximity, is not convenient or safe. In this work, we propose a long-range “zero interception” ambient backscatter (LoRAB) communication system relying on low power sensor (tag) deployments. Without employing a dedicated radio transmission, our technology enables the “zero interception” communication of the tags with portable receivers over hundreds of meters. This enables low-cost and low-power communications across a wide range of missions by using chirp spread spectrum (CSS) modulation on ambient FM signals. A laboratory prototype exploiting commercial components (laptops, DAQ, software-defined radios (SDR) platform) have demonstrated the potential by achieving 130 m tag-to-reader distance for a low bit rate of 88 bps with the modulator current consumption at around 103 nA.

## 1. Introduction

Radio signals are a suitable technology for remote communication. However, the present state-of-the-art entails an active transmitter either at the node/tag (e.g., Bluetooth, ZigBee, LoRa or equivalent), at the reader (e.g., radio-frequency identification (RFID) or equivalent) or both ends (e.g., WiFi, 4G LTE, 5G NR or equivalent). The active transmission of radio signals is traceable using relatively simple spectrum equipment, and therefore communication based on techniques relying on aforementioned principles can be easily detected (it is not suitable for security applications). The spatial distribution of the signal strength can further reveal the location of the transmitter. In addition, when it comes to distributed sensor nodes, the radio transmission may consume over 99% of the overall power budget, posing the need for batteries.

Low-power and low-complexity backscatter communications (e.g., RFID) have emerged as a promising paradigm to address the aforementioned challenge of power consumption. While passive nodes are within today’s state-of-the-art, the range of these systems is short (typically well below 10 m) and this can be impractical in applications where approaching the node at such a proximity is not convenient or safe. Moreover, although in these systems the node is not transmitting radio signals, an active transmitter is deployed in the reader that makes the entire system liable to detection. This work is targeting three technological challenges:

**Technological challenge 1**: Long range ambient backscattering communications. In order to remove the need for active radio transmission, the system exploits available radio frequency (RF) signals in the environment to modulate and transfer information between the node and the reader [1]. These types of systems are suitable for defense and security applications where the sensor node is not an active device. The detection of these signals is virtually impossible without prior knowledge of their existence.

While the capability to modulate information using the backscattering of ambient signals has been demonstrated theoretically and experimentally [2,3,4] by our team, the range of communication within the state-of-the-art is of the order comparable to commercial RFID systems, i.e., below 10 m. The core technological challenge along the envisioned plan, which is addressed in this paper, relates to extending the range of ambient backscattering communication systems by at least an order of magnitude, to several 100 m. By virtue of the operating principle, the signal that carries information from the node to the reader in the envisioned system is at very low power levels that can be below the ambient noise floor. Consequently, the system is practically hard to detect and neutralize.

**Technological challenge 2**: Duty-cycling power autonomous operation. By virtue of eliminating active radio transmission, sensor nodes will operate on energy harvested by the environment (e.g., solar, RF harnessing or both) [5]. In order to enable power autonomy, a second technological challenge relates to the development of a microwatt system with a power management system that exploits a sleep and wake-up duty-cycling together with a supercapacitor that is charged by a small photovoltaic cell (or tiny photodiode) and the rectification of ambient RF signals. Significantly, duty-cycling dispenses with the need for any bespoke (and therefore detectable) wake-up signals to activate the tags.

**Technological challenge 3**: Software defined radio (SDR) implementation of the receiver compatible with consumer electronics devices. The versatility of the system is further underpinned by the flexible implementation of the readers in widely available SDR receivers, such as those available in consumer electronics hand-held devices (e.g., smartphones). The third technological challenge to be addressed entails the development of a hand-held receiver implemented on a smartphone.

This work aims to enable covert “zero interception” wireless communications from battery-less power autonomous sensor nodes that dispense the need for dedicated radio-frequency emission. This is achieved by bridging the gap between the following Internet of Things (IoT) technologies:

1. Battery-less RFID tags, which typically require a bespoke reader signal;
2. Battery operated Long Range (LoRa) sensors, which require battery and emit detectable radio signals;
3. Recently emerged ambient backscattering communications, which enable communication by virtue of adjusting the scattering of ambient RF signals at the tag’s antenna.

On one hand, the above technologies 1 and 2 would require active/dedicated RF transmission (TX), which therefore make the system liable to detection and being power hungry. On the other hand, most previous demonstrations [2,6,7] of ambient backscattering are limited to ranges of a few meters (typically less than 30 m), which can be impractical for real life applications.

**Figure 1:**
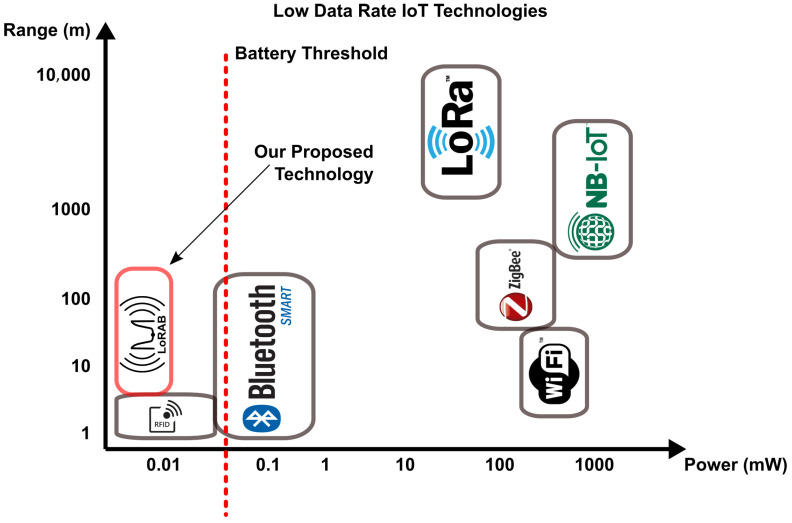
Our proposed technology compared to the currently existing Internet of Things (IoT) technologies.

The key innovation we introduce relates to the exploitation of chirp spread spectrum (CSS) modulation in ambient backscattering communications. As is depicted in Figure 1, our proposed technology, next termed **LoRAB** (**Lo**ng **R**ange **A**mbient **B**ackscatter), covers the gap of battery-free and long range IoT sensors and the choice of broadcast FM radio signals exploits that the required infrastructure already exists in the rural and urban areas worldwide.

The proposed CSS modulation scheme, which is commonly used in LoRa IoT communications, is known to provide excellent correlation properties compared to the other modulations schemes, thereby substantially increasing the communication range, while also spreading the power of the information carrying signal over a finite bandwidth. Previous works [8,9] have demonstrated backscattering systems capable of generating a linear frequency chirp signal modulated by properly varying the phase of a reflected wave. With the constraint of an incoming carrier wave signal, both works relied on an active transmission from a dedicated TX, which eliminates the benefits of ambient backscattering concept. In another recent work [10], the authors implemented an FM band backscatter using a tag amplifier with the tunnel diode. As the tunnel diode works with “negative resistance”, the modulator consumes a large enough power around 90 μW with a 23 dB gain over the FM bandwidth. According to their tests, they can achieve 20 m tag-to-reader range under an FM signal strength of −50 dBm. The tag-to-transmitter (FM station) range was around 4.7 Km.

Our work demonstrates a 6–7× superior distance between the tag and reader using an ultra-low-power modulator with power consumption in the order of nanoWatts. Another novel aspect of this work is that it is taking advantage of the existing infrastructure of the FM band and the backscattered signals can be decoded using a modified commercial FM receiver.

In breadboard implementation involving a commercial off-the-shelf DAQ controlled by a laptop and a single RF transistor as well as a fully implemented SDR receiver, we have demonstrated real-time data rates of 125 Bps over a range of 130 m. This demonstrator validated the operating principle and capabilities of LoRaB and CSS ambient backscattering (critical function and characteristic proof-of-concept/basic validation in a laboratory environment).

The paper is organized as follows: Section 2 provides the basic principles of backscatter communication and Section 3 presents previous works with short range ambient backscattering. Section 4 presents the principle of the long range “zero interception” ambient backscatter and Section 5 presents the design of the proof-of-concept system prototype. Section 6 shows the communication testing results, Section 7 presents the envisioned plan and Section 8 the main conclusions.

## 2. Backscatter Communications

**Figure 2:**
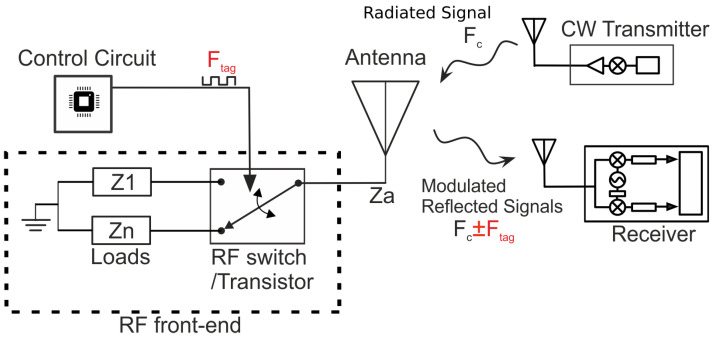
The emitter transmits a carrier signal and the tag reflects a small amount of the approaching signal back to the reader. The tag modulates the backscattered signal by changing the load connected to its antenna terminals resulting in a $\Gamma_{i}$ change.

Backscatter communication typically uses RFID tags. A general RFID system includes three devices: a backscatter node (i.e., a tag), a receiver (RX) and a transmitter (TX). The tag receives a continuous wave (CW) signal and scatters a fraction of it back to the RX (Figure 2). The communication can be implemented by reflecting and modulating the incoming signal. The RF front-end part of the tag typically consists of a single RF transistor or a switch connected with an antenna. By virtue of changing the impedance load connected to the antenna terminal, and thereby the resulting reflection coefficient, the tag superimposes the information on the incoming CW. More specifically, the tag modulates the reflected signal using a specific modulation scheme. Conventionally, the reader-to-tag communication is based on conventional Amplitude/Phase shift Keying (ASK/PSK) modulation schemes. For example, in backscatter binary modulation, the reflected signal is modulated by switching the load between two discrete values ($Z_{1}$ and $Z_{2}$), resulting in two reflection coefficients ($\Gamma_{1}$ and $\Gamma_{2}$):

(1) $$\begin{array}{c} \Gamma_{1/2}=\frac{Z_{1/2}-Z_{a}^{*}}{Z_{1/2}+Z_{a}}, \end{array}$$

with $Z_{a}$, the antenna impedance of 50 Ohm.

Amplitude-shift keying (ASK) modulation constitutes the basic operation of commercial RFID systems. Using two reflection coefficients, it is also possible to achieve FSK modulation, whereby subcarriers appear next to the carrier with frequencies $F_{c}\pm F_{tag}$, where $F_{c}$ represents the carrier frequency and $F_{tag}$ the frequency of the load modulation. This approach is presented in our previous work [11] and provides improved sensitivity at the increase of complexity and power consumption. As depicted in Figure 2, the modulator (control unit) of the tag controls the transistor with a signal that has frequency $F_{tag}$.

## 3. Ambient Backscatter Communications

Commercial RFID systems commonly utilize monostatic architectures where the reader consists of both the TX that emits the wave needed for backscatter communication and the RX that decodes the tag-modulated signals. Bistatic architectures, dislocating the TX from the RX, offer a more flexible topology and an increased tag-to-RX communication range [12]. Examples of bistatic backscatter setups are presented in our previous works [11,13].

Ambient backscattering is an idea based on the bistatic backscatter approach that utilizes ambient signals for backscattering. This simplifies the communication scheme since it eliminates the need for TX part. In earlier works [2,4] we have demonstrated system implementations operating in an on-off keying (OOK) modulation scheme and four level pulse amplitude modulation (4-PAM) on a modulated FM broadcast radio signal. The choice of broadcast FM radio exploits that such infrastructure already exists globally. However, the concept is applicable to different ambient signals such as ambient TV, Cellular or WiFi signals. In our case, we focus on FM radio signals as this is ubiquitously present.

Our heritage implementation [2] relied on fully integrated tags based on a 16-bit microcontroller (MCU) powered from a 0.1 F supercapacitor. The tag also includes a real-time clock (RTC) to wake up the MCU from the “sleep” operation mode, where the current consumption of the board is 0.02μA. The MCU generated 50% duty cycle pulses that control an RF switch, thus generating an OOK modulated backscattered signal. The current consumption at 1 MHz was 126μA at 2.3 V (290μA). The MCU has a 12-bit analog-to-digital converter (ADC) which was used to read analog output signals from sensors. The demonstrator also involved a real-time SDR receiver implemented on a low cost (22 USD) RTL; SDR was used as RX. With this setup we have demonstrated the capability to transmit OOK data up to 2.5 KBps over distances of up to 5 m. The prototype tag was tested in a real-time indoor laboratory deployment with a real FM station at 34.5 Km away from the tag (RX-to-tag distance). A video of the initial demonstrator is available here: Vimeo Video, https://vimeo.com/228836739 (accessed on 1 May 2022).

A full implementation along similar lines was used to demonstrate our 4-PAM concept involving a tag powered exclusively from harvested solar energy [4]. The proposed system was improved in terms of power consumption and demonstrated in an indoor environment with a low bit rate of 345 bps and power consumption only 27μW. In all occasions our demonstrator included a full packet definition and the system can also be augmented with error correction coding. The tags are also operating with all the FM stations.

## 4. Long-Range Ambient Backscatter

**Figure 3:**
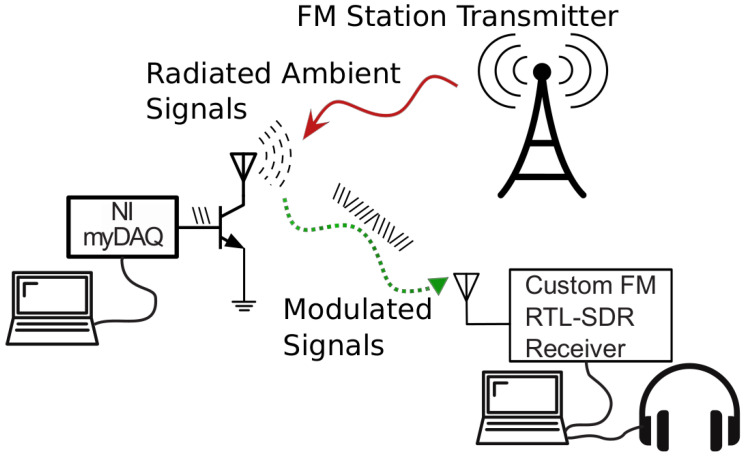
Long range backscatter communication is achieved using ambient FM signals. A custom FM receiver was designed for the reception of the the chirp spread spectrum modulated backscattered signals.

In this work, we consider CSS modulation of the backscattered signals and we demonstrate the first proof-of-consent prototype suitable for long-range ambient FM backscatter communications (Figure 3). The TX transmits a music signal and the tag reflects and modulates (using chirp signals) a fraction of the approaching signal back to the reader (RX). The tag modulates the backscattered signal by changing the load connected to its antenna terminals resulting in reflection coefficient ($\Gamma_{i}$) changes.

The CSS modulation scheme, which is used in Long Range (LoRa) IoT communications, is known to provide excellent correlation properties, thereby substantially increasing the communication range, while also spreading the power of the information carrying signal over a finite bandwidth. In our previous work [8], we demonstrate backscattering systems capable of generating a linear frequency modulated by properly varying the phase of a reflected wave instead of directly varying its instantaneous frequency. The work exploits a bespoke RF front-end involving two RF transistors that generate a set of impedances by changing their gate bias. It is numerically and experimentally demonstrated that this circuit can backscatter an incoming continuous wave (CW) as a chirp spread spectrum signal. To validate the proposed design, several LoRa symbols were generated using a two-channel arbitrary waveform generator (AWG) and were successfully transmitted and decoded. With the constraint of an incoming CW signal, the work in [8] relied on an active transmission from a dedicated TX, which removes the benefits of ambient backscattering.

A key characteristic of CSS is that a time delay in a chirp signal translates to a frequency shift at the output of the fast Fourier transform (FFT). CSS modulation uses this to encode data (sequences of bits) as cyclic time shifts in the baseline chirp. Four parameters are used to define our custom chirp signal:

1. the bandwidth (BW);
2. the spreading factor (SF);
3. the starting frequency ($F_{\mathrm{start}}$);
4. the coding rate (CR).

The chirp’s linear frequency sweep is equivalent with the spectral bandwidth (BW) of the chirp and the symbol rate (SR) is calculated as:

(2) $$\begin{array}{c} SR=\frac{BW}{2^{SF}} \end{array}$$

symbols per second. Since each chirp symbol can represent SF bits and the bit rate (BR) is calculated as:

(3) $$\begin{array}{c} BR=\frac{SF*SR*4}{\left(4+CR\right)}. \end{array}$$

**Figure 4:**
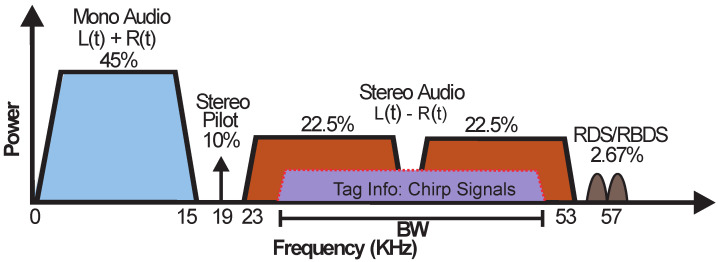
Baseband Spectrum of a generic modern-day FM audio station. For our proposed CSS modulation, the chirp signals are placed within the stereo FM radio spectrum.

In order to achieve CSS backscattering, we exploit the specific characteristics of the baseband signal spectrum used in FM radio broadcasting, illustrated in Figure 4. The FM station modulates the audio stream in mono mode by encoding the sum of the left and right audio channels (L + R). In stereo mode, the audio stream is encoding in the L − R area.

The proposed tag can generate chirp signals with frequencies covering the L + R, the L − R (Figure 4) or the whole spectrum area of the FM station. In cases of the L + R and L − R areas, the signal can be received by a commercial FM receiver or a smartphone with FM capabilities. In our proof-of-concept case, the receiver was implemented on a software-defined radio (SDR) platform. Alternatively, several commercially available smartphones include conventional FM receivers that could also be used to implement it. Scanning the environment, and selecting the FM station with the strongest received power, could further increase the tag-to-RX maximum range.

## 5. System Design

**Figure 5:**
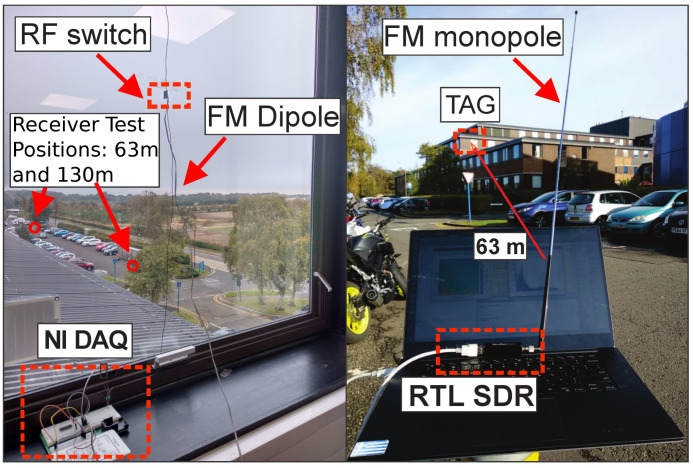
The long range ambient backscatter setup, comprising the tag (**left**) indoors and the receiver in an outdoors environment (**right**).

To demonstrate our proposed topology, we put together a full prototype system (tag and custom receiver) demonstrating the capability of CSS ambient backscattering signals to produce long-range communications. In our prototype implementation, the tag consists of a commercial digital-to-analog converter (DAC) unit (NI myDAQ) connected with an RF front-end that includes a wired dipole antenna for FM signal reception/reflection and an RF switch for the modulation. The implemented RF front-end is similar with work [2]. The DAC is controlled by a laptop through MATLAB or Python. The chirps signals produced by the DAC (Figure 5, left) drive the RF switch on the RF front-end.

A tag encoding algorithm was developed on the tag side and was programmed to transmit a fixed packet of chirp signals with modulation parameters SF=9, BW=10 kHz, $F_{start}=43$ kHz and CR=4. As it is observed, the $F_{stop}=F_{start}+BW=53$ kHz and the chirp signals cover only one stereo subband of the FM spectrum. All the above parameters were selected in order to optimize the packet error rate and the total time duration of the packet transmission. This allows any FM stereo receiver to tune to the stereo mode and receive the backscatter-generated signals by subtracting the L channel from the R channel. At the beginning, a bitstream of useful information (transmitted data) is translated to a packet of chirp symbols. Each packet includes the symbols of the useful information which is defined as payload, plus extra symbols that are useful for the reception procedure (preamble symbols). In order to increase the communication efficiency, the bitstream of useful information was encoded before it is converted to chirp symbols. For the encoding we used the techniques of:

1. Hamming (8, 4) encoding;
2. interleaving operation;
3. Gray indexing.

Hamming encoding is defined by the factor CR and is very useful as it is able to effectively detect double bit errors and correct single bit errors.

In the RF front-end, the ADG919 RF switch was chosen due to its ultra-low-power consumption. Its power consumption follows the equation:

(4) $$\begin{array}{c} \frac{1}{2}*C_{\mathrm{RF}}V_{DD}^{2}F_{\mathrm{sw}}, \end{array}$$

which is the CMOS dynamic consumption. The $F_{\mathrm{sw}}$ is the maximum switching frequency and $C_{\mathrm{RF}}$ the dynamic power dissipation capacitance at RF path when it is ON. For $F_{\mathrm{sw}}=53$ KHz, $V_{DD}=1.8$ V, and $C_{\mathrm{RF}}=1.2$ pF (@ 1 MHz) the power consumption of the switch was estimated at 185 nW (103 nA). The 185 nW refers to the front-end only and the overall consumption of the tag should include the power dissipated in the DAC, which can be drastically minimized by its replacement with a system-in-package (SiP) implementation of the tag as discussed in the conclusion.

The receiver consists of a low-cost RTL SDR (Figure 5, right) that downconverts the received RF signal to baseband and sends in-phase and quadrature samples to the GNU-Radio framework through a USB interface.

**Figure 6:**
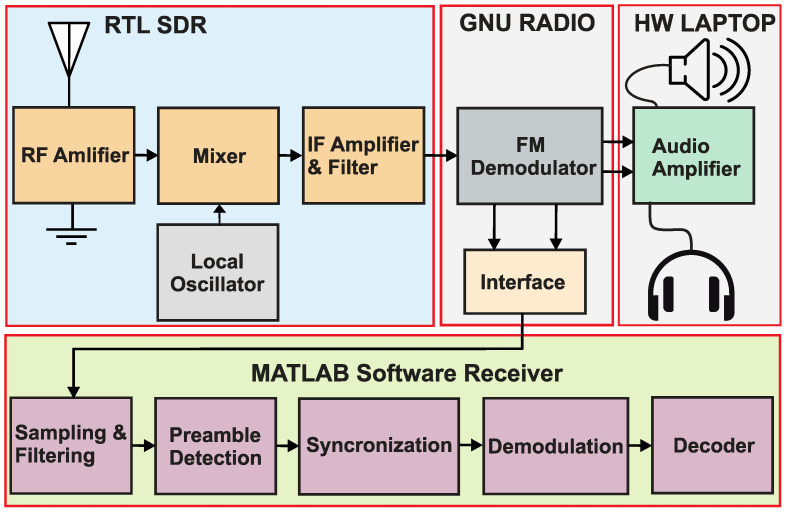
Proposed implementation of the FM based receiver.

The implementation of the reader is depicted in Figure 6. The GNU Radio Companion is GNU Radio’s graphical tool that implements digital signal processing using visual depictions known as flowgraphs. A Linux computer is required and the GNU-Radio supplies the MATLAB software with samples through a first-in first-out (FIFO) buffer for real-time signal processing. In GNU-Radio, we implemented a flow-graph to receive stereophonic FM broadcasts. FM radio implementation consists of

1. a signal source—in our case, RTL-SDR dongle;
2. a low pass filter;
3. a wideband FM (WBFM) demodulator block;
4. an audio output—PC’s sound card.

The WBFM demodulator accomplishes the tasks necessary to receive FM stereo. It separates and demodulates the L + R and L − R signals and then it performs the multiplexing to separate them into the L and R stereo channels, before sending them to an audio sink block to make the sound audible on the PC’s speakers. For our proof-of-concept demonstration, we need the L − R, so an interface block was implemented and configured to subtract L and R signals and provide the result as an output signal. The interface block pushes the data to FIFO and MATLAB reads them for the demodulation procedure. The MATLAB implementation includes down-sampling of the signal, preamble detection, synchronization and demodulation∖decoding of the payload.

**Figure 7:**
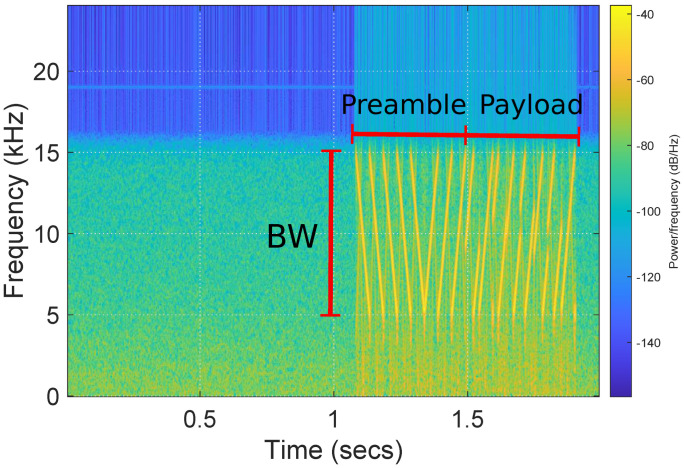
Received signal spectrogram including a packet of cyclic frequency chirp signals.

The spectrogram of Figure 7 depicts the received packet after down-sampling. The figure shows a sequence of five repeating down-chirps and three up-chirps at the beginning that represent the preamble. The preamble is followed by the encoded payload. The number and the type of preamble symbols were fixed after experiments and the payload was a fixed bit-stream known at the receiver side. The binary information modulates the slope of the linear cyclic chirps, with up-chirps (positive slope) and down-chirps (negative slope). The receiver demodulates these symbols by multiplying the incoming signal with a baseline chirp and then performs FFT. Since multiplication in the time domain is a correlation in the frequency domain, the receiving operation results in a peak in the FFT frequency bin corresponding to the time delay in the received chirp.

## 6. Communication Testing Results

**Figure 8:**
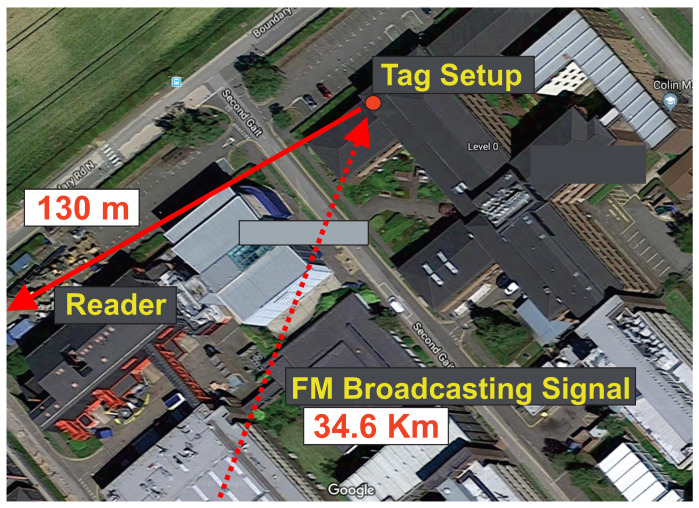
Tag-Rx range measurement setup. The tag antenna was placed 130 m away from the receiver antenna and the FM station located 34.6 Km away.

In order to perform tag-to-receiver range measurements, our system was tested indoors/outdoors using the most powerful FM station that was measured in the area. The RF front-end antenna was placed on the window (Figure 5, left) while the FM station antenna (at 98.5 MHz) was 34.6 Km away from the measurement’s setup Earl Mountbatten building, Heriot-Watt University: https://goo.gl/maps/wNw9eqZ7T9hGe4Rx9 (accessed on 1 May 2022) (Figure 8). The radiated power of the FM antenna was around 250 KW and the power of the received FM signal next to the tag antenna, was measured at around −45 dBm. Our university area does not have line of sight to the FM station antenna and thus the received signals are multipath in nature and are low in signal strength [14]. The tag, and the receiver (RX) were tested in fixed tag-to-RX outdoor distances of 63 m and 130 m. The bit rate of the communication can be calculated as 87.89 bps and an experimental analysis of the error rate performance will be provided as a future work.

We could consider a system with multiple antennas at the tag and receiver side [15,16,17] as a future work in order to decrease the packet error rate and increase the communication distance. A non-coherent detector could be provided based on the proposed channel model of [15]. Simulation results in [15] showed that an enhanced bit error rate performance can be achieved.

A successful wireless communication was achieved and it was observed that, when tag-to-receiver distance decreases, the reader can successfully decode more packets. It was also observed that the indoor object attachments and multi-path fading have significant effects on the performance of the system especially in terms of the communication range and bitrate between the tag and the receiver. The above observation can be confirmed from the indoor range results of our previous work [4]. In this work the measurements were performed outdoors with a line-of-sight between the tag and the reader, so multi-path fading may have less significant effects.

Our developed system belongs to the category of RFID tags, so it is not illegal under current rules. However, the reflected FM signals could interfere with commercial FM receivers. For this reason, a simple experiment with an FM receiver nearby shows that there is no interference at all, but a detailed experimental study is required in the future to determine the level and limits of interference generated by our ambient backscatter system.

## 7. Future Plans

This demonstration was used to validate the operating principle and capabilities of FM CSS ambient backscattering (critical function and characteristic proof-of-concept validation in a laboratory environment), but it does not meet form-factor (volume/mass/size) or power-consumption requirements. The prototype uses a commercial lab instrument (NI-myDAQ) that is power hungry/costly and it is not optimised for our design. As a future work aiming at miniaturized and more power-efficient implementations (eliminating the power consumption of the DAC), we plan to build a system-in-package (SiP) implementation of the tag. The main component will be an FPGA that receives the data from a sensor and controls a single transistor for the ambient backscattering. The SiP will also include the oscillators (main oscillator and real time clock), the power circuit and a small flexible solar panel in order to charge a super capacitor and make the system completely battery-free.

The preferred implementation in order to achieve the maximum level of integration and power efficiencies, is a System on Chip (SoC) approach in a customized application-specific integrated circuit (ASIC). This approach allows us to customize both the digital modulator and the core such that the entire system can run on a low clock speed of some MHz, resulting in power consumption that we estimate to be in the order of 10 microWatts. The ASIC will also include a rectifier that will enable the system to operate on harvested RF energy from WiFi, FM, and TV signals that can power the system (albeit at a slower rate) in the absence of solar energy. It is noted that RF harvesters [18,19] are now able to power ultra-low-power MCUs such that it is indeed possible to envision total independence of the system from solar energy. In addition to maximizing performance as per above, the SoC implementation will further optimize the integration leading to a very compact system. A single ASIC will integrate the following functionalities: RF front-end, digital modulation, power management and system control. This will leave only the antenna, the preferred sensor and the supercap to be integrated out of the package together with the optional solar panels for additional powering.

## 8. Conclusions

In this paper, a novel approach for long-range “zero interception” ambient backscatter tag was presented. The main advantages we foresee in our technology are:

(i) The lack of any active RF transmitter. Our system relies on very weak backscatter signatures of otherwise existing ambient signals. Additionally, the use of spread spectrum modulation makes the detection of these signals virtually impossible without prior knowledge of their existence;
(ii) An integrated bespoke ASIC that provides all necessary analogue and digital circuitry is anticipated to consume a uW level of power. Consequently, for relatively moderate duty cycle operations it is possible to power the nodes using either a very small photodiode or an RF rectifier charging a supercapacitor through a power management circuit. This provides a very long operation life while enabling very compact packaging of the node.

In a proof-of-concept breadboard implementation involving a commercial off-the-shelf DAC, controlled by a laptop and a single RF transistor switch as well as a fully implemented SDR receiver, we have demonstrated real-time data rates of 88 bps over tag-to-Rx ranges up to 130 m. Our approach could be useful for future agricultural sensing applications, as the monitoring of plants require low data rates and this is of high importance for smart agriculture.

## 9. Patent

Spyridon-Nektarios Daskalakis and George Goussetis, Long range ambient backscatter apparatus, WO2022013572A3, filed 16 July 2021 and issued 20 January 2022.

## Data Availability

Not applicable.
